# Association of Reading Comprehension and Science Aptitude with Early Success in a First-Semester BSN Cohort: A Cross-Sectional Study

**DOI:** 10.3390/nursrep15100363

**Published:** 2025-10-10

**Authors:** Marivic B. Torregosa, Orlando Patricio

**Affiliations:** 1 Department of Nursing, Texas A&M International University, Laredo, TX 78041, USA; 2 Department of Natural Sciences, Laredo College, Laredo, TX 78040, USA; orlando.patricio@laredo.edu

**Keywords:** students, nursing, reading, comprehension, educational measurement, program evaluation, cross-sectional studies

## Abstract

**Background**: As the United States population becomes increasingly diverse, the representation of minorities in health professions is critical to addressing health disparities. Few investigations have been conducted among students enrolled in the first semester of the nursing program, a vulnerable and adjustment period for most nursing majors. Thus, this study examined the association between reading comprehension and science aptitude on student retention and standardized test scores. **Method**: A cross-sectional repeated measures study was conducted to investigate the outcomes from a compendium of programmatic interventions implemented among *n* = 80 nursing students enrolled in the first semester of a pre-licensure baccalaureate nursing program in one Hispanic-serving institution. These interventions included the Weaver™ reading online program, case studies, NCLEX-type practice tests, test-taking skills, and peer-mentoring. Data collection was conducted in Spring 2024. Multivariate statistical analysis was used to determine predictors associated with student retention and standardized test scores. An independent *t*-test was used to examine any significant difference in the reading comprehension level among the cohort’s participants. A qualitative investigation using thematic analysis was conducted to understand students’ experiences with the programmatic interventions. **Results**: Students’ baseline reding comprehension level was significantly associated with failure in the first semester of the nursing program (*β* = −0.815; *SE* = 0.349; Wald = 5.444; *p* < 0.05). End-of-term reading comprehension level was significantly associated with end-of-course HESI score in the Foundations in Nursing course (*β* = 26.768; *SE* = 10.049; Beta = 0.445; *p* < 0.05) while science GPA was significantly associated with end-of-course HESI score for Health Assessment (*β* = 3.022; *SE* = 1.315; Beta = 0.434; *p* < 0.05. Cohort retention was 75%. The independent *t*-test result indicated a significant difference in reading level was found between those who dropped out from the cohort (*M* = 4.23, *SE* = 0.173 and those who did not (*M* = 5.15, *SE* = 0.188), *t* (68) = −3.037, *p* < 0.01. A reading level of grade 10 and above was associated with student progression to the next semester (*M* = 10.16, *SE* = 0.375, *t* (70) = −0.560, *p* < 0.05. Although the participants found the reading comprehension modules tedious, test-taking strategies, applying the nursing process in case studies, and the expertise of a nurse educator, who understood the learning needs of first-semester students, were perceived as critical to academic success. **Conclusions**: Reading comprehension and science aptitude are essential to students’ early success in the nursing program. Addressing gaps in reading comprehension and science aptitude before admission to a nursing program would increase chances of success in the early stages of a nursing major.

## 1. Introduction

As the United States population becomes increasingly diverse, the representation of minorities in health professions is critical to addressing health disparities [1]. While the Health Resources Services Administration estimates a sufficient nursing workforce by 2030, shortages will still be observed at the regional and local levels, especially in medically underserved areas [2,3]. Although the state of Texas estimates a 30.5% increase in the nursing workforce by 2030, the nursing demand will remain high at 38.8% [4]. More specifically, a healthcare workforce that reflects the increasingly diverse patient population nurses serve is critical to addressing the healthcare gap [1]. One way to address the nursing shortage is to increase the student success and retention rates of nursing students who are in the early stages of a nursing major.

There are many reasons for the nursing shortage. Reports indicate that students from minority backgrounds face many barriers to academic success in nursing [5,6,7,8,9]. Language barriers were detrimental to students’ reading comprehension, communication, completing assignments, test-taking skills, and test performance in standardized tests [6,9,10]. Poor test-taking and reading skills and test anxiety all lead to course failure, partly due to English being a second language and the inability to handle the reading load required in nursing courses [11,12,13]. In addition, financial barriers and family issues relating to balancing academic and family obligations do not facilitate success [14,15]. Similarly, lack of student integration into the learning environment and perceptions of bias and discrimination in the learning environment contributed to low retention [5,8,16]. Nursing programs are under pressure to maintain accreditation. In Texas, nursing programs must maintain an 80% pass rate on the NCLEX (National Council Licensure Examination) on the first attempt to remain in good standing with the state board of nursing. High retention rates, on-time graduation, and high NCLEX pass rates are some benchmarks nursing programs must meet to maintain accreditation. Thus, nursing programs have created admission policies to identify students who are likely to be successful in a nursing major [8,17,18,19] and implement programmatic interventions to increase student retention and success.

Programmatic interventions to improve student success in nursing programs have been implemented in past studies. These include peer mentoring [10,20,21]; faculty mentoring [21,22]; stipends and tuition reimbursement to offset financial barriers [8,22]; social and academic support in the learning environment such as remediation, translation, writing workshops, retention specialist [15,20,23,24,25,26]; NCLEX examination preparation strategies [21,26,27]; and flexible scheduling and additional time with assignments [22,24]. While retention strategies have been reported in the literature to assist at-risk students, few intervention studies have been conducted among students who are in the early stages of a nursing major [24,26]. The most challenging period for a nursing major is in the first semester after admission into the program, as this is the period of adjustment to the clinical and didactic demands of a nursing program. Also, students may assume and underestimate that the academic efforts exerted in pre-nursing courses will still be effective in passing nursing courses. It is essential to understand the impact of programmatic interventions in the early stages of a nursing major to promote student retention and on-time graduation and therefore address the nursing shortage. A robust nursing workforce will be impossible to achieve if students are not successful at the early stages of the nursing program. Thus, it is critical to examine the effectiveness of programmatic strategies implemented in the early periods of the nursing program.

The main aim of the study was to examine whether a reading comprehension intervention implemented in a nursing course was associated with student retention and test scores in standardized tests. The secondary aim was to gather students’ perceptions of the programmatic interventions.

## 2. Theoretical Framework

Tinto’s theory on persistence was used as a theoretical framework for the programmatic interventions implemented in this study [28,29]. Originally developed in 1975, Tinto’s Longitudinal Model of Institutional Departure drew its basic premises from Durkheim’s theory of suicide and Van Gennep’s rite of passage theory [30]. Durkheim’s theory indicates that departure from society or “egotistical suicide” could occur if one fails to integrate and assimilate into one’s community. On the other hand, Van Gennep noted that as a person moves from one place or stage to another, specific rites of passage occur. The celebration of these rites of passage is a symbol of a person’s integration and acceptance in their new environment. The anthropological nature of Van Gennep’s and the sociological perspective of Durkheim’s theory provided the foundation for Tinto’s central tenet that students depart from college prematurely when they fail to integrate into the university environment [30]. He posits that students drop out of the institution when they need to be more integrated or collectively affiliated. As students progress through college, they interact with an academic and social system that will significantly impact their academic development. Students’ social and academic experiences will have some bearing on integration into the learning environment [31]. The more socially and academically integrated students feel towards the learning environment, the more they are committed to their goals and remain in college. Tinto’s thesis concerns how students fit into the college environment and how their perceptions of their relationships with peers, faculty, and staff will significantly impact social and academic integration. Failure to realize the above conditions will result in student departure from the institution [28]. Tinto’s theory on persistence served as the guiding framework in the selection of the interventions, as we assumed that the mechanism of the strategies implemented in this study led to positive academic and social experiences that could mitigate students’ early departure from their major.

## 3. Methods

This study was conducted in a traditional prelicensure BSN program offered in one Hispanic-serving institution located on the border between Mexico and the United States (US). Students are admitted into the program at the junior level (first semester) after completing college core courses. After admission, it takes four semesters to complete the program. Overall, the university student body is 97% Hispanic. The student body of the BSN program mirrors that of the university. The participants of this study were a cohort of nursing students, *n* = 80, who were newly admitted into the nursing program and were enrolled in nursing courses offered in the first semester of the major.

A repeated measures cross-sectional investigation was conducted to examine whether a programmatic intervention on reading had any positive impact on student retention and standardized test scores. Retention is defined as the number of students who successfully passed the first semester courses and remained on track with their cohort. Students who were not retained within the cohort were considered “drop/fail”. Drop from cohort was coded (yes = 1; no = 0). Test scores from standardized tests, HESI™ (Health Education Systems Incorporated), in Foundations in Nursing and the Health Assessment nursing courses were collected at the end of the 15-week term.

A compendium of programmatic interventions was implemented in this study to operationalize social and academic integration (Table 1). Weaver™, a web-based reading program, was implemented because a prior analysis of existing institutional data in the BSN program indicated that students’ reading levels predicted attrition [32]. The reading software is a comprehensive online computer-based instructional program that provides students with extensive assessment, instruction, and practice in the areas of vocabulary, comprehension, and fluency. The software is self-paced and is appropriate for students from the early elementary grades through post-secondary. The vocabulary component of the software incorporates 7000 core vocabulary words published by the Educational Development Laboratory (*EDL Core Vocabularies*), based on the idea that new words are best learned through repetitive usage and exposure [33]. Initially, an assessment is given, and based on the assessment results, assignments are tailored to match the reader’s reading level. Activity logs and students’ progress are recorded in the portal that are both visible to the faculty and the student. The software determines mastery and advances the reader to the next level. The reading assignments become complex as the reader’s reading comprehension advances. A license for Weaver was purchased for each participant. The investigators declared no conflict of interest with Weaver™. The validity of Weaver™ is provided by MetaMetrics, also known as the Lexile Framework for Reading [34]. Repeated measures on reading scores were taken at baseline and at the end of the 15-week term [35]. Weaver™ has been used in a statewide study of 27 prelicensure nursing programs in Texas; however, reading scores were not linked to standardized test scores [36].

Other interventions that were implemented to promote academic integration included case studies, test-taking strategies, and critical thinking taught by a seasoned nursing faculty (Table 1). An NCLEX workbook was provided to learn test-taking strategies and critical thinking. Informal peer-to-peer mentoring was implemented between junior and senior nursing students to enhance social integration into the learning environment. Snacks were served during these informal mentoring meetings to encourage attendance.

The first author deployed an online survey using open-ended questions to collect students’ perceptions about the interventions implemented in the program. The open-ended questions included [1] *Please describe what aspects of the course you found most helpful,* [2] *Please describe which areas of the course you found least helpful.* Response rate was 54%. Data collection was conducted from January 2024 through May 2024. The use of online surveys for qualitative research is common in other disciplines [37,38]. Students’ perceptions about the programmatic interventions were grouped into themes manually using Braun and Clarke’s thematic analysis method [39]. The first author is quite familiar with Braun and Clarke’s method of thematic analysis [39] based on her past work [40,41]. Data analysis was conducted by hand in six steps:

1. This step involved reading and rereading the narratives to obtain familiarity and a broad understanding of the data.
2. Codes were generated manually after familiarization with the data. Data extracts or quotes were matched to each code.
3. The generated codes and their matching data extracts were sorted and examined for patterns or themes.
4. Similar themes collapsed. Data extracts were re-examined to determine the suitability of the identified theme. If data extracts did not match the theme, these were relocated to other themes, or a new theme was developed. Coding and recoding were ongoing until the data extracts, codes, and themes fit together.
5. The essence of each theme was examined for its fit to the overall aim of the qualitative aspect of this investigation.
6. The relation among themes was explored.

The themes were discussed informally with participants after the term ended to avoid a sense of coercion. Approval from the university ethics board was obtained before the study was conducted. Participation in the programmatic interventions implied consent; however, the participants were instructed that if they wanted their data excluded from the analysis, the investigators would readily remove them. No participants indicated exclusion of their data.

## 4. Data Analysis

Descriptive statistics were conducted to achieve a general overview of the data. Multivariate statistical analyses were conducted to determine which predictors were associated with retention and end-of-course HESI standardized test scores. A Kolmogorov–Smirnov (KS) test for normality was conducted for all continuous variables in the model. The KS statistic for overall GPA, Science GPA, overall HESI score, and HESI score for Health Assessment were not significant (*p* > 0.05), indicating the normal distribution of errors [42,43]. The rest of the variables were not normally distributed. No data transformation and adjustments were made to the datasets or for the analyses for multiple comparisons, as the sample size used in this study was considered high enough to detect a significant effect. Cases with missing values were excluded from the analysis.

An independent *t*-test was used to examine any significant differences in the reading level among those retained in the cohort. A *p*-value of 0.05 was considered significant. Nursing program admission data, including pre-nursing GPA, Science GPA, and HESI A2 scores for Anatomy and Physiology, Grammar, Math, Reading, Vocabulary, HESI A2 overall scores, Weaver™ reading scores, and end-of-course HESI scores in Foundations in Nursing and Health Assessment, were treated as continuous variables.

## 5. Results

At the end of the term, the cohort’s retention rate was 75% compared to past semesters’ 50% to 60%. Students’ mean grade point average (GPA) on admission was 3.58 (Table 2). The mean science GPA on admission was 3.54. Students’ mean comprehensive reading level at baseline was 4.857; the minimum was 3; the maximum was 7. Students’ mean score for reading from the HESI A2 admission test was 86.525. A score of 75 was considered acceptable for admission into the program. The average reading comprehension recorded on Weaver™ at the end of the 15-week term was grade 10.042. The average end-of-course HESI score for the Foundations in Nursing course was 863.104, and 921.984 for the Health Assessment course. A score of 850 on the end-of-course HESI was considered acceptable in the program.

Science GPA was positively correlated with overall admission GPA (*r* = 0.671; *p* < 0.01). Attrition was negatively associated with overall admission GPA (*r* = −0.258; *p* < 0.05); science GPA (*r* = −0.339; *p* < 0.01); baseline reading comprehension level (*r* = −0.346; *p* < 0.01); Foundations in Nursing course grade (*r* = −0.795; *p* < 0.01); Health Assessment course grade (*r* = −0.626; *p* < 0.01); and Pharmacology course grade (*r* = −0.310; *p* < 0.05). The negative correlations above imply that attrition in the first semester could be mitigated by requiring a high science GPA and high reading comprehension level upon admission, and high grades in first semester courses such as Foundations in Nursing, Health Assessment, and Pharmacology. Admission GPA was positively associated with Foundations in Nursing course grade (*r* = 0.414; *p* < 0.01), Health Assessment course grade (*r* = 0.452; *p* < 0.01), and Pharmacology course grade (*r* = 0.396; *p* < 0.01) (Table 3). These positive correlations suggest that a high GPA requirement for program admission could lead to high grades in Foundations in Nursing, Health Assessment, and Pharmacology. An independent *t*-test indicated a significant difference in reading level was found between those who dropped out from the cohort (*M* = 4.23, *SE* = 0.173 and those who did not (*M* = 5.15, *SE* = 0.188), *t* (68) = −3.037, *p* < 0.01. A reading level of grade 10 and above was associated with student progression to the next semester (*M* = 10.16, *SE* = 0.375, *t* (70) = −0.560, *p* < 0.05. Students’ baseline reading comprehension level predicted being off-track or drop from the cohort (*β* = −0.815; *SE* = 0.349; Wald = 5.444; *p* < 0.05) (Table 4). This means that while holding all other variables constant, a 1-unit increase in baseline reading level is associated with a 55.7% decrease in the odds of being off-track (drop/fail) from the cohort. The result indicates that a low baseline reading comprehension level could result in attrition in the first semester of the nursing program. Reading comprehension level at the end of the semester predicted the end-of-course HESI score in the Foundations in Nursing course (*β* = 26.768; *SE* = 10.049; Beta = 0.445; *p* < 0.05) (Table 5). Science GPA predicted end-of-course HESI score for Health Assessment (*β* = 3.022; *SE* = 1.315; Beta = 0.434; *p* < 0.05 (Table 6). The participants spent an average of 26.5 h on Weaver™.

### 5.1. Descriptions of the Lived Experience Perceived as Most Helpful

***Rehearsing practice questions.*** Practice sessions on test-taking helped students learn how to select an answer among closely related choices in nursing examinations. The exercise allowed them to read, reflect, and look for what was being asked in the test question. By gaining test-taking strategies, students could see progress in their test performance in nursing courses across the semester (Table 7).

The test-taking practice helped a lot in learning to prioritize choices in tests.

I actually stopped to look at them and read what was being asked.

*It helped me better understand how to tackle my nursing exam questions as well as assignments.*

*Doing the practice care plan was extremely helpful in doing my care plan as well; I do not think I would have done as well as I did without the samples we practiced in the seminar. It has been great to sit in class and notice the progress I have made from the beginning of the semester to this point. I have noticed a significant change in the way I think.*

***Obtaining a bigger picture.*** Case studies and the nursing process helped students develop critical thinking and a better understanding of the concepts presented in nursing courses.

*Case studies helped me to see the bigger picture.*

*I believe the case studies were a great resource and helped improve my critical thinking.*

*The course helped me develop a better understanding of the nursing process.*

***Knowledgeable faculty.*** Student success in gaining test-taking skills and critical thinking in analyzing test questions can be attributed to the expertise of an experienced faculty member.

*The nursing faculty was very knowledgeable and helped me develop critical thinking.*

*The course helped me develop to think like a nurse and dissect the questions and applied in on my course exams.*

***Delayed appreciation.*** Gaining test-taking skills and critical thinking will take some time to develop, especially for first-semester nursing students who did not encounter NCLEX-type questions in previous courses prior to a nursing major. Slow progress may lead to frustrations; however, once students see improved test scores in nursing courses, appreciation for such initiatives follows.

*What I found least helpful was that in the first weeks of this class, I felt like it was not helpful, and I didn’t pick up on any strategies until the last four weeks.*

*It has been great to sit in class and notice the progress I have made from the beginning of the semester to this point.*

### 5.2. Descriptions of the Lived Experience Perceived as Least Helpful

***Time-consuming reading.*** Students found the reading modules time-consuming in addition to the demands of other nursing courses. They felt overwhelmed with added reading assignments. It is possible that students were not accustomed to the reading load required in nursing and health science majors.

*The Weaver reading program was my least favorite part. It was time-consuming since the coursework was already heavy.*

***Peer-mentoring.*** Forging a meeting between the first semester and senior nursing students to promote peer mentoring was viewed as not helpful. In these meetings, senior nursing students shared tips on how to be successful in the program. These informal gatherings in which food was served did not assist in establishing networks for first-semester nursing students in the program.

*Meeting with seniors was the least helpful.*

*It was a waste of time.*

***Class timing.*** Students found the programmatic interventions an added burden due to competing demands from other nursing courses. The timing schedule of the class influenced on student’s interest in the programmatic interventions.

*The class is long and scheduled at the end of the day. I would have liked the class to be in the morning when we were fresh and not tired.*

*We were taking four classes already.*

## 6. Discussion

This study examined the association of reading comprehension with student retention and end-of-course standardized test scores in nursing courses. This study found that end-of-semester reading comprehension was associated with student retention in the first semester of the nursing program and scores on standardized tests. Our finding corroborates past findings on the significant impact of reading comprehension on student retention in the nursing program [7,32,36]. The current study findings extend the science in this field by providing evidence on the link between reading comprehension and student retention at the early stage of the nursing program and scores on standardized tests. Past studies cite reading comprehension’s critical role in achieving academic success in the nursing program [7,8,11,32]. The current finding implies that nursing program applicants’ reading comprehension levels need to be assessed before program admission to increase their likelihood of success in the early stages of the major. Nursing courses require heavy reading to understand nursing concepts; standardized tests require the test taker to understand vocabulary to analyze test questions critically. Thus, poor reading comprehension skills could lead to poor test performance from test anxiety, which then could lead to course failure. The current finding supports Tinto’s theory that academic integration, in this case, reading comprehension ability, prevents the premature departure of students from the academic institution.

The current finding suggests that standardized admission tests used in nursing programs may not be sensitive to screen for reading comprehension deficiencies; thus, it is recommended that reading comprehension be assessed for all college freshmen, ESL (English as a second language), and international students entering college. Administrators in higher education may need to invest in developing infrastructures to identify reading gaps. With the advent of dual credit and college credit transfers, quality control on reading comprehension skills is essential, as general education courses are now being taught in high schools and later transferred and accepted at institutions of higher learning. College students’ reading gaps should be identified and addressed before admission into a major such as nursing.

A strong association between improved reading comprehension and standardized test scores in first-semester nursing courses was found in the current study. Past studies found that reading is the strongest predictor for student success and, more specifically, success in courses offered in the first semester of the nursing program [7,11,32]. The current findings corroborate past studies citing the strong influence of science aptitude on course grades, early nursing certification examination [44], and performance in standardized tests [17,18,45]. Investigators in nursing education have recommended that science aptitude be prioritized among nursing program admission requirements [11,18]. Other studies found that science aptitude was significant in determining students’ success in nursing program completion and passing the NCLEX-RN [15,17,45]. Students who have gained a solid foundation in anatomy and physiology may have greater ease in understanding concepts presented in nursing and medical textbooks. Knowledge in the sciences is then retrieved and applied in case studies and scenarios presented in nursing tests and clinical practicums. Addressing weaknesses in the sciences at the pre-nursing level is recommended to enhance success in the nursing program. Nursing faculty and program administrators may need to collaborate with the science departments to examine the content taught in science courses to prepare students for a nursing major. In addition, collaborating with high school academic advisors, counselors, and science teachers is essential to enhance science aptitude among high school students who want to major in nursing, medical, and health sciences.

While improved reading comprehension had a significant relationship with retention, students found the reading modules time-consuming and the least helpful. The participants were concurrently enrolled in first-semester courses, and completing the self-paced reading modules was considered a burden. On the other hand, practice sessions focused on analyzing NCLEX-type questions and the nursing process in approaching case scenarios helped students develop critical thinking.

Programmatic interventions, such as those conducted in this study, may only be successful if they are taught by an expert nurse educator who understands the learning needs of students at this stage in the nursing program. Therefore, it is recommended that nursing programs invest in the professional development of nursing faculty so they can grow into becoming expert nurse educators and develop teaching skills to facilitate the academic success of the future generation of nurses.

Although students initially complained about the relevance of the programmatic strategies implemented in the current study, student buy-in only occurred once they saw improvement in their test scores. This implies that students in this stage of the nursing program can easily get frustrated when previously learned study skills no longer work in nursing program coursework. Therefore, students at this stage of the program need to be mentored to develop resilience to cope and adjust to the demands of the major. Resilience in handling stress and the demands of a nursing major would serve students well in the long term as they face the challenges in the program and the real world of the nursing profession.

This study attempted to implement unstructured mentoring by pairing the first semester with senior nursing students to operationalize Tinto’s tenet on social integration into the learning environment. However, such an intervention was not successful due to class schedule conflicts. Structured peer-to-peer mentoring activities are recommended in future studies. Incentivizing mentoring program participants may result in observable positive outcomes. A mentoring advisor may be needed to guide the mentoring activities.

## 7. Conclusions

Improved reading comprehension and science aptitude are critical to early success in the nursing program and academic integration into the learning environment. Thus, reading comprehension ability should be assessed for all college entrants with demographic characteristics similar to the current study’s sample. Addressing reading comprehension gaps and science aptitude deficiencies before students enter a nursing major would increase their success in the early semesters of the nursing program. Reinforcing science aptitude among students who are pursuing careers in nursing, medicine, and health science should begin in primary and secondary education. Nursing programs must collaborate with K-12 science teachers to inform a curriculum that will prepare students for these careers.

The geographic location, the sample and the sample size, and research design used in this study limit the generalizability of the current study findings. We recommend replicating the current study in large and diverse samples and in other geographic locations using longitudinal research designs. Only the reading comprehension intervention was quantitatively measured in terms of outcomes; other interventions were measured anecdotally. Psychological and socio-cultural factors that may impact student success in the first semester of the nursing program were not accounted for; thus, we recommend including these in future investigations. Social integration, as posited in Tintos’ theory, needs a more in-depth analysis.

This study adds to the current literature by providing information on the critical need to identify and address reading comprehension gaps for all nursing program entrants. The current finding confirms past studies on the role of reading comprehension and science aptitude in student success in nursing. The programmatic interventions implemented in this study could be scaled in other nursing programs. With the nursing shortage and the requirement to pass standardized tests such as the NCLEX for entry into practice, improved reading comprehension skills and science aptitude are necessary to address the nursing shortage.

## Figures and Tables

**Table 1:** Academic and Social Integration interventions.

Interventions for academic integration across the 15-week semester	Case studies; Weaver™ reading comprehension vocabulary and medical terminology modules x 15 weeks; Next Generation NCLEX Case Studies and Test Questions practice tests; NCLEX workbook was provided; a seasoned nurse educator was assigned to the course.
Interventions for social integration across the 15-week semester	Informal peer-mentoring with senior nursing students Snacks were provided during peer mentoring sessions to encourage attendance.
Measures	Retention rate of the cohort; test scores on HESI™ standardized tests; Weaver™ reading scores.

**Table 2:** Descriptive data.

Variables	Min	Max	Mean	SE	SD
Overall GPA ^1^	3.21	4	3.581	2.274	20.340
Science GPA ^2^	3.08	4	3.543	2.854	25.524
HESI Anatomy & Physiology ^3^	76	100	92.800	0.673	6.018
HESI Grammar ^4^	78	98	89.850	0.594	5.313
HESI Math ^5^	76	98	91.075	0.604	5.400
HESI Reading ^6^	76	98	86.525	0.434	3.881
HESI Vocabulary ^7^	76	100	91.975	0.694	6.207
HESI Overall Score ^8^	80	96	90.438	0.399	3.564
Baseline Reading Level ^9^	3	7	4.857	0.149	1.243
Hours on Weaver ^10^	0.854	102.471	26.514	2.369	20.103
Current Reading Level ^11^	4	12	10.042	0.317	2.688
Foundations in Nursing Course Grade ^12^	0	3	1.450	0.107	0.953
Health Assessment Course Grade ^13^	0	3	1.900	0.117	0.980
Pharmacology Course Grade ^14^	0	3	1.896	0.074	0.606
Drop/Fail ^15^	0	1	0.275	0.050	0.449
HESI Score in Foundations in Nursing ^16^	95	1201	863.104	20.361	166.661
HESI Score in Health Assessment ^17^	550	1343	921.984	22.043	173.564

^1^ Overall pre-nursing GPA upon program admission. ^2^ Science GPA. ^3^ HESI entrance exam score Anatomy and Physiology. ^4^ HESI entrance exam score for Grammar. ^5^ HESI entrance exam score for Math. ^6^ HESI entrance exam score for Reading. ^7^ HESI entrance exam score for Vocabulary. ^8^ HESI entrance exam overall score ^9^ Baseline reading level on Weaver™. ^10^ Hours spent on Weaver™. ^11^ Reading level at the end of the 15-week term on Weaver™. ^12^ End of term course grade for Foundations in Nursing (A = 3; B = 2; W/F = 0). ^13^ End of term course grade for Health Assessment (A = 3; B = 2; W/F = 0). ^14^ End of term course grade for Pharmacology (A = 3; B = 2; W/F = 0). ^15^ Drop/Fail Students not on track with cohort due to course withdrawal or course failure (Yes = 1; No = 0). ^16^ End of term HESI score for Foundations in Nursing. ^17^ End of term HESI score for Health Assessment.

**Table 3:** Correlation of variables.

Variables	1	2	3	4	5	6	7	8	9	10	11	12	13	14	15	16	17
1 Overall GPA	1																
2 Science GPA	0.671 **	1															
3 HESI Anatomy and Physiology	−0.122	0.052	1														
4 HESI Grammar	−0.115	−0.036	0.340 **	1													
5 HESI Math	0.261 *	0.270 *	0.141	0.277 *	1												
6 HESI Reading	0.272 *	0.210	0.036	0.127	0.393 **	1											
7 HESI Vocabulary	−0.116	0.035	0.364 **	0.603 **	0.306 **	0.158	1										
8 HESI Overall Score	0.015	0.147	0.590 **	0.733 **	0.645 **	0.433 **	0.792 **	1									
9 Baseline Reading Level	0.111	0.263 *	0.031	−0.054	0.026	0.005	−0.036	−0.013	1								
10 Hours on Weaver	0.006	0.024	−0.030	−0.109	−0.020	0.002	0.028	−0.034	0.007	1							
11Current Reading Level	0.091	0.110	−0.125	−0.158	0.190	−0.011	−0.155	−0.074	0.249 *	0.449 **	1						
12 Foundations in Nursing Course Grade	0.414 **	0.435 **	0.016	−0.051	0.097	0.093	−0.156	−0.014	0.347 **	−0.019	0.168	1					
13 Health Assessment Course Grade	0.452 **	0.492 **	0.037	−0.085	0.111	0.184	−0.123	0.020	0.268 *	0.070	0.163	0.693 **	1				
14 Pharmacology Course Grade	0.396 **	0.275 *	0.004	−0.069	0.070	0.175	0.028	0.052	0.132	0.101	0.079	0.422 **	0.618 **	1			
15 Drop/Fail	−0.258 *	−0.339 **	−0.007	0.028	−0.029	0.032	0.066	0.027	−0.346 **	0.028	−0.067	−0.795 **	−0.626 **	−0.310 *	1		
16 HESI Score in Foundations in Nursing	0.152	0.237	0.038	−0.081	−0.021	−0.179	−0.050	−0.083	0.244	0.055	0.367 **	0.400 **	0.313 *	−0.114	−0.578 **	1	
17 HESI Score in Health Assessment	0.268 *	0.366 **	0.002	0.123	0.299 *	0.141	0.081	0.197	−0.074	−0.095	−0.049	0.439 **	0.653 **	0.383 **	−0.291 *	0.269 *	1

* *p* < 0.05; ** *p* < 0.01.

**Table 4:** Predictors for Drop or Fail in the first semester.

Variables	B	S.E.	Wald	Sig.	Exp(B)	95% C.I. for EXP(B)	
						Lower	Upper
Overall HESI Score ^1^	−0.072	0.348	0.042		0.931	0.471	1.841
Overall GPA ^2^	−0.022	0.022	0.987		0.979	0.938	1.021
Science GPA ^3^	−0.021	0.016	1.858		0.979	0.949	1.009
HESI Anatomy and Physiology ^4^	0.029	0.103	0.076		1.029	0.840	1.260
HESI Reading ^5^	0.119	0.141	0.712		1.127	0.854	1.487
HESI Vocabulary ^6^	0.012	0.108	0.012		1.012	0.819	1.250
Time Spent on Weaver™ ^7^	0.003	0.017	0.034		1.003	0.970	1.038
HESI Grammar ^8^	0.006	0.110	0.003		1.006	0.810	1.249
Baseline Reading Level ^9^	−0.815	0.349	5.444	*	0.443	0.223	0.878
Current Reading level ^10^	0.015	0.139	0.012		1.015	0.773	1.333
Constant	9.810	11.623	0.712		18,214.783		

* *p* < 0.05; Nagelkerke *R*^2^ = 0.328. Cox and Snell *R*^2^ = 0.234. Homer and Lemeshow χ^2^ (8) = 3.331; *p* = 0.722. The Area Under the Curve (AUC) Receiver Operating Characteristic (ROC) Curve = 0.865. This is an excellent range. There is an 86.5% chance that the model will correctly distinguish a randomly chosen positive case from a randomly negative case. The model is very good at predicting the outcome (drop/fail). ^1^ Overall score on the HESI entrance exam. ^2^ Overall pre-Nursing GPA prior to admission into the nursing program. ^3^ Science GPA. ^4^ HESI score for Anatomy and Physiology. ^5^ HESI score for Reading. ^6^ HESI score for Vocabulary. ^7^ Time spent on the Weaver™ reading program. ^8^ HESI score for Grammar. ^9^ Baseline reading level as measured by Weaver™ reading program. ^10^ Current reading level at the end of the 15-week term using the Weaver™ reading program.

**Table 5:** Predictors of Foundation in Nursing HESI Score.

Variables	B	SE	Beta	Sig
(Constant)	963.950	725.731		
Overall GPA ^1^	0.901	1.569	0.111	
Science GPA ^2^	1.383	1.271	0.199	
HESI Anatomy and Physiology ^3^	−1.409	16.342	−0.053	
HESI Grammar ^4^	−2.610	16.717	−0.084	
HESI Math ^5^	−7.038	18.108	−0.231	
HESI Reading ^6^	−9.874	18.542	−0.244	
HESI Vocabulary ^7^	3.478	18.013	0.120	
HESI Overall ^8^	3.601	83.709	0.073	
Baseline Reading Level ^9^	10.423	17.313	0.082	
Current Reading Level ^10^	26.768	10.049	0.445	*
Hours on Weaver ^11^	−1.533	1.1493	−0.198	

* *p* < 0.05; *R*^2^ = 0.293. *Adjusted R*^2^ = 0.121. ^1^ Overall pre-nursing GPA prior to admission into the nursing program. ^2^ Science GPA. ^3^ HESI entrance exam score for Anatomy and Physiology. ^4^ HESI entrance exam score for Grammar. ^5^ HESI entrance exam score for Math. ^6^ HESI entrance exam score for Reading. ^7^ HESI entrance exam score for Vocabulary. ^8^ HESI entrance exam overall score. ^9^ Baseline reading level on Weaver™. ^10^ Reading level on Weaver™ at the end of the 15-week term. ^11^ Hours spent on Weaver™ reading program.

**Table 6:** Predictors of Health Assessment HESI Score.

Variables	B	SE	Beta	Sig
(Constant)	569.896	728.116		
Overall GPA ^1^	−1.122	1.678	−0.129	
Science GPA ^2^	3.022	1.315	0.434	*
HESI Anatomy and Physiology ^3^	−18.185	18.558	−0.696	
HESI Grammar ^4^	−14.940	18.929	−0.476	
HESI Math ^5^	−8.180	21.136	−0.264	
HESI Reading ^6^	−18.861	20.687	−0.456	
HESI Vocabulary ^7^	−22.794	22.219	−0.721	
HESI Overall Score ^8^	82.255	96.579	1.697	
Baseline Reading Level ^9^	−13.449	18.561	−0.103	
Current Reading Level ^10^	−18.919	11.515	−0.291	
Hours on Weaver ^11^	0.132	1.230	0.016	

* *p* < 0.05; *R*^2^ = 0.229. *Adjusted R*^2^ = 0.044. ^1^ Overall pre-nursing GPA upon admission into the nursing program. ^2^ Science GPA. ^3^ HESI entrance exam score for Anatomy and Physiology. ^4^ HESI entrance exam score for Grammar. ^5^ HESI entrance exam score for Math. ^6^ HESI entrance exam score for Reading. ^7^ HESI entrance exam score for Vocabulary. ^8^ HESI entrance exam overall score. ^9^ Baseline reading level in Weaver™. ^10^ Reading level at the end of the 15-week term on Weaver™. ^11^ Hours spend on Weaver™.

**Table 7:** Students’ qualitative descriptions regarding strategies.

Most Helpful	Least Helpful
**Rehearsing practice questions** *Test taking practice helped a lot; learning to prioritize choices in tests.* *I actually stopped to look at them and read what was being asked.* *Practice with the bowtie was helpful.* *It helped me better understand how to tackle my nursing exam questions as well as assignments. Doing the practice care plan was extremely helpful in doing my care plan as well; I do not think I would have done as well as I did without the samples we practiced in seminar. It has been great to sit in class and notice the progress I have made from the beginning of the semester to this point. I have noticed a significant change in the way I think.*	**Time consuming reading** *Weaver reading program was my least favorite part. It was time consuming since the coursework was already heavy.*
**Obtaining a bigger picture** *Case studies helped me to see the bigger picture.* *I believe the case studies were a great resource and helped improve my critical thinking.* *The course helped me develop a better understanding of the nursing process.*	**Peer-mentoring** *Meeting with seniors was the least helpful.* *It was a waste of time.*
**Knowledgeable faculty** *The nursing faculty was very knowledgeable and helped me develop critical thinking.* *The course helped me develop to think like a nurse and dissect the questions and applied in on my course exams.*	**Class timing** *The class being long and scheduled at the end of the day. I would have liked the class to be in the morning when were fresh and not tired.* *We were taking four classes already.*
**Delayed appreciation** *What I found least helpful was that in the first weeks of this class I felt like it was not helpful, and I didn’t pick up on any strategies until the last four weeks.* *It has been great to sit in class and notice the progress I have made from the beginning of the semester to this point.*	

## Data Availability

The data presented in this study are available on request from the corresponding author due to an ongoing longitudinal tracking of participant outcomes from the interventions conducted in this study.

## Public Involvement Statement

No public involvement in any aspect of this research.

## Guidelines and Standards Statement

This manuscript was drafted against the Strengthening the Reporting of Observational Studies in Epidemiology (STROBE) for cross-sectional studies.
